# Patient safety education at Japanese nursing schools: results of a nationwide survey

**DOI:** 10.1186/1756-0500-4-416

**Published:** 2011-10-17

**Authors:** Shoichi Maeda, Etsuko Kamishiraki, Jay Starkey, Kazumasa Ehara

**Affiliations:** 1 Graduate School of Health Management, Keio University, 4411 Endo, Fujisawa, Kanagawa, 252-8530, Japan; 2 Graduate School of Social Welfare, University of Kochi, 2751-1 Ike, Kochi City, Kochi, 781-8515, Japan; 3 University of California, San Diego, Dept. of Internal Medicine, 200 W. Arbor Drive # 8425, San Diego, CA 92103-8425, USA; 4 Graduate School of Health Care Sciences, Jikei Institute, 1-2-8 Miyahara Yodogawa-ku Osaka, 532-0003, Japan

## Abstract

**Background:**

Patient safety education is becoming of worldwide interest and concern in the field of healthcare, particularly in the field of nursing. However, as elsewhere, little is known about the extent to which nursing schools have adopted patient safety education into their curricula. We conducted a nationwide survey to characterize patient safety education at nursing schools in Japan.

**Results:**

Response rate was 43% overall. Ninety percent of nursing schools have integrated the topic of patient safety education into their curricula. However, 30% reported devoting less than five hours to the topic. All schools use lecture based teaching methods while few used others, such as role playing. Topics related to medical error theory are widely taught, e.g. human factors and theories & models (Swiss Cheese Model, Heinrich's Law) while relatively few schools cover practical topics related to error analysis such as root cause analysis.

**Conclusions:**

Most nursing schools in Japan cover the topic of patient safety, but the number of hours devoted is modest and teaching methods are suboptimal. Even so, national inclusion of patient safety education is a worthy, achievable goal.

## Background

In recent years, medical error has gained increasing attention from the medical community and public at large. The demand to improve the safety of healthcare has never been higher [1-4]. Safety education during professional school is becoming an important means to achieve greater patient safety [5-8]. Nursing staff are integral to patient safety and nursing education has become a focus of recent efforts [9-15]. Notably the WHO is currently working on a multi-professional patient safety curriculum guide in partnership with the International Council of Nurses and others [16].

### The importance of patient safety

Beauchamp & Childress identified the "four principles in medical ethics": 1) respect for patient autonomy, 2) beneficence, 3) non-maleficence, and 4) justice [17]. Patient safety is important with regards to medical ethics in the sense of non-maleficence. A "World Alliance for Patient Safety" article published by WHO in 2005[18] reported data on adverse events in health care from several countries and concluded that too many patients suffer from preventable harm and adverse effects. Japan is no exception [2].

From a societal standpoint, patient safety is paramount. Monetarily, for example, medical error harms the public. For example, in the UK additional hospital stays are estimated to cost about £2000 million a year and in the USA preventable adverse medical events, including lost income, disability and medical expenses, is estimated to cost many millions per year, not to mention the erosion of trust, confidence and satisfaction among the public and health-care providers [18]. Because Japan has been less open about medical error and its costs until recently, the exact cost to Japan is unknown; nevertheless, the toll of medical error on society in Japan is likely high.

### The importance of patient safety education at nursing schools

Nurses are central to the issue of patient safety because they are often the provider with the most direct contact and sustained care with any given patient. According to the Japanese "Project to Collect Medical Near-Miss/Adverse Event Information 2009 Annual Report" by the Japan Council for Quality Health Care, the number of medical accidents involving nurses accounts for half of the total medical accidents each year [19]. Appropriately, nursing education in regards to patient safety has been a focus of improvement for medical error prevention.

To understand how patient safety education fits into the nursing curriculum, a brief introduction to Japanese nursing education is in order. Several routes to nursing licensure in Japan are available. Compulsory foundational nursing education is first provided at 4-year colleges and universities, 3-year junior colleges, or 3-year vocational schools. Nursing educational institutions are regulated under different authorities: colleges, universities, and junior colleges are under the jurisdiction of Ministry of Education, Culture, Sports, Science and Technology (MEXT) while most vocational schools are under the jurisdiction of Ministry of Health, Labour and Welfare (MHLW) [20]. After completing nursing school, prospective nurses must then pass a national licensing examination before practicing. Education about medical safety is left to the discretion of each educational institution.

### A national standardized nursing curriculum guideline

Interest in adopting patient safety education into the nursing curriculum appeared around 2004 when the Japanese Ministry of Internal Affairs and Communications published a series of brief reports regarding the curricula of 11 university-affiliated nursing schools [21]. A committee was formed to determine the "current state of nursing education" and the conclusions of the committee included a need for "safety skills", though no specific details were provided. The Ministry of Education formed a committee in 2009 to develop a national standardized nursing curriculum guideline, similar to that which had already been created for medical school education [22]. The committee is to formulate and publish the guideline by the end of 2011. While the guideline is not finalized at the time of this publication, early recommendations include some items related to patient safety [23].

### Subject of this research

Despite the recent attention to patient safety education in nursing curricula, the current state of patient safety education at nursing schools in Japan is unknown. We therefore aim to determine current patient safety educational practices regarding how much, by what instructional methods, and about what topics nursing schools teach patient safety. This information may aid in decisions regarding resource allocation and strategy for improving patient safety education in Japan and provide information to the international community about what is achievable in terms of adopting safety education in nursing curricula.

## Methods

This is a cross-sectional research study. We developed a structured, anonymous, self-administered survey consisting of 7 items with multiple sub-responses regarding patient safety education and a final section regarding school characteristics. We based the questionnaire on the current WHO guidelines [16], the Japanese model core curriculum guidelines for patient safety education [22] and our previous works regarding to the management of adverse events [24].

In Japan, government guidelines regarding human subjects research specify that this type of survey research does not require IRB approval or written informed consent [25]. However, we explained our research thoroughly in the cover letter, stated that participation was voluntary, and asked that participants only fill out the questionnaire if they understood and consented to participation, essentially providing the equivalent of written informed consent. Our research was compliant with the Helsinki Declaration.

The survey was mailed via the Japanese postal system to all 193 public and private Japanese university-affiliated nursing schools in operation as of April 2010. The list of Japanese nursing schools was obtained from the MEXT website [26].

It is of note that, as mentioned above, non-university institutions also provide nursing education [20]. We chose to focus on university-affiliated nursing schools because these schools set the standard for nursing education and because no comprehensive listing of non-university affiliated schools exists. Surveys were addressed to the dean of each school for distribution to the professor in charge of patient safety education. Data collection occurred from April 1st to 15th, 2010. We used JMP8.0 software for statistical analysis. We compared the data for public schools and private schools using chi-squared analysis, unless the expected frequency for a cell was less than five, in which case we used Fisher's exact test. We used the Mann-Whitney U test for analyzing class hours. Significance was set at an alpha less than 0.05 and statistically significant differences between public and private nursing schools are denoted by †.

## Results

### Participation (Table 1)

**Table 1:** Responses to a 2010 National Survey of Safety Education at Japanese Nursing Schools

	Public	Private	Total
Number of eligible schools (n)	89	104	193

Student enrollment			
(Average)	72.7	86.5	80.2
(Maximum)	120	200	200
(Minimum)	40	40	40
(median)	80	80	80

Respondents (n)	43	35	83*

Participation rate (%)	48.3	33.7	43.0

* 5 schools returned a survey but did not indicate if they were public or private.

Out of 193 nursing schools, we received a completed questionnaire from professors in charge of nursing education at 83 schools, for a participant rate of 43%. Fifty-five-percent of the respondents were from public universities and 45% were from private universities.

### Patient safety curricular inclusion

Seventy-five (90%) of the respondents indicated their schools cover the topic of patient safety in any form. Thirty-two (45%) devoted courses specifically to patient safety.

### Total hours (Figure 1)

**Figure 1:**
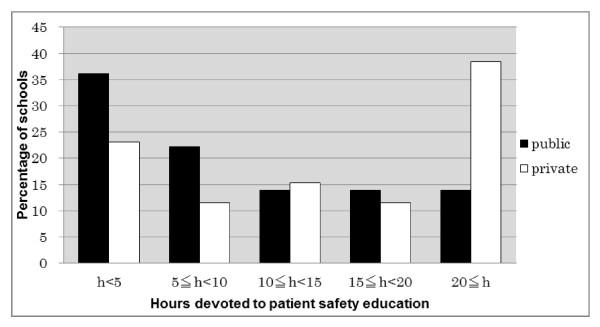
**Hours Devoted to Safety Education as Reported by Participants in the 2010 National Survey of Safety Education at Japanese Public and Private Nursing Schools**. Private schools devoted statistically significantly more hours to patient safety education (p = 0.02). For statistical analysis, the Mann-Whitney U test was used; blank responses were excluded.

Public and private nursing schools in Japan devoted on average 9.43 (SD ± 7.65) and 14.73 (SD ± 9.9) total curricular hours to patient safety education, respectively. Outlier data were excluded from analysis. Private schools devoted statistically significantly more total curricular hours compared to public schools (p = 0.02). Thirty-percent of nursing schools devoted less than five hours.

### Teaching methods (Table 2)

**Table 2:** Teaching Methods Utilized for Safety Education as Reported by Participants in the 2010 National Survey of Safety Education at Japanese Nursing Schools

School Type	Public (N = 41)	Private (N = 29)	Total (N = 70)
	n (%)	n (%)	n (%)
Lecture	41(100.0)	29(100.0)	70(100.0)
Group discussion	23(56.1)	14(48.3)	37(52.9)
Student presentations	8(19.5)	4(13.8)	12(17.1)
Field trips	9(22.0)	2(6.9)	11(15.7)
Role play	6(14.6)	2(6.9)	8(11.4)
Simulations	2(4.9)	2(6.9)	4(5.7)
Others	5(12.2)	3(10.3)	8(11.4)

For statistical analysis, the chi-square test or Fisher's exact test was used; blank responses were excluded.

There were no significant differences between public and private nursing schools.

All schools taught on the topic of patient safety through lectures and few employed other methods such as role-play. Thirty-seven (52.9%) of nursing schools used group discussions. Public and private schools did not differ in reported methods of teaching.

### Patient safety topics (Additional file 1)

Patient Safety Education Topics by Category and Topic were examined as shown in Additional file 1. Topics covered by more than three quarters of schools included theories and models of error, human factors, verifying patient identity, double-checking, communication with senior stuffs, and criminal liability. Less than one quarter of schools covered failure mode and effects analysis, sharing adverse events with other institutions for learning, reporting unnatural deaths to the police, or recommending autopsy (Additional file 1 Management of adverse events) and all topics related to autopsy (Additional file 1 Autopsy).

When compared to public schools, statistically greater private schools covered the topic of root cause analysis while coverage of other topics did not differ significantly.

## Discussion

### Patient safety curricular inclusion

Ninety percent of respondents reported that their nursing school has incorporated some form of patient safety education into the curricula. Still, 10% of respondents reported having no patient safety education. This is unacceptably high. Given the tendency of selection bias of survey research and the good subject effect, it is likely that we have selected for schools that have already incorporated topics on patient safety into their curricula; if so, the true rate of nursing schools incorporating patient safety is probably even lower.

### Total hours (Figure 1)

Most schools devoted more than 5 hours to patient safety and some schools more than 20. Private schools reported devoting more time to patient safety education than public schools. However, the overall participation rate was lower for private schools, and this difference in time devoted may be overestimated. In any case, if acquisition of knowledge and skills to maximize patient safety requires adequate exposure, and acquisition of such skills leads to increased patient safety, it is paramount that nursing students receive adequate exposure to this topic. While it would be impractical to definitively prove that safety education increases patient safety, it makes sense that such education would improve patient safety [27]. Schools should devote more time to the topic.

With regards to how much time needs to be devoted, it is first necessary to establish what topics need to be covered. In 2009, the WHO published the "Patient Safety Curriculum Guide for Medical Schools" [28]. In Japan, The Japanese Ministry of Education also has published a guideline for medical education called the Model Core Curriculum (MCC) that was revised in 2008 to include patient safety as part of the core medical curriculum [22]. This medical school core curriculum focuses on the prevention of error. However, because errors will happen [4], an important part of patient safety is responding to adverse events [24,29], for example, the concepts of apology, management of medical personnel following an adverse event, and autopsy (Additional file 1). We think that it is important to incorporate these topics into any future curricular guidelines. The WHO is updating their guideline to include input from the areas of dentistry, midwifery, nursing, pharmacy and related health-care professions with the aim of developing a multi-professional edition to inform, support and assist the inclusion of patient safety in the curricula of all health professionals [16]. The Japanese government is planning to develop guidelines for nursing schools in Japan, too [30]. As these guidelines are created, the number of topics that need to be covered is expanding. The subjects we included in our survey would be a reasonable array of topics to cover in a basic patient safety curriculum. We believe that at least one educational unit, defined in Japan as 15 periods of 90 minutes, or 22.5 hours of education time, would be required to minimally cover these topics.

### Teaching methods (Table 2)

Traditional lecture-based education has been heavily employed in many educational settings because of the efficiency in mass information transmission while using few resources in terms of educators, preparation time, and classroom space. All schools that teach patient safety use lecture-based methods. Yet, other teaching methods such as role-playing are probably more effective in training students to apply the theoretical and practical skills in real life settings [10,12]. About 50% of nursing schools use group discussions as a means of enhancing the "skills" part of education. We suggest nursing schools explore other teaching methods to increase the quality of education on safety.

### Patient safety topics (Additional file 1)

#### Topics covered by more than three quarters of schools

Topics covered by more than three quarters of schools are theories and models of error, human factors, verifying patient identity, double-checking, communication with senior stuffs and criminal liability.

In Japan, two serious medical accidents occurred in 1999: the Yokohama City University Hospital case (Jan. 1999) and the Hiroo General Hospital case (Feb. 1999). These cases became national impetus for patient safety improvement and nursing errors were major contributors to patient harm in both cases. In the Hiroo case, for example, a nurse administered an antiseptic (chlorhexidine) intravenously. The nurse mistook it for heparin sodium after another nurse had left it on the cart. The patient died immediately. The case received national media attention, prompting police involvement. In the wake of these cases, investigators emphasized the need for education on theories and models of error, human factors contributing to error, and practical error prevention strategies like verifying patient identity and double checking. Consequently, these topics have been incorporated into the curricula of more than three quarters of schools. The Hiroo case was a sentinel case handled through the Japanese criminal legal system, and subsequent cases of medical error have been handled likewise; prior research has shown the total number of healthcare provider criminal prosecutions for medical error leading to patient death has been on the rise for over 10 years [31]. This may be why so many nursing schools cover the topics of criminal liability.

#### Topics covered by less than one quarter of schools

Less than one quarter of schools covered reporting unnatural patient deaths to the police, autopsy, cross-institutional data sharing for error prevention, or failure mode and effects analysis.

In Japan, physicians are currently required to report healthcare-associated patient deaths to the police under the Japanese Medical Practitioner's Law. Article 21 of the law states, "In the course of pronouncing death of any person or fetus over the age of 4 months should the physician find anything unnatural, he or she must report that death to the police within 24 hours." Therefore, Japanese physicians grapple much with how to handle patient death in the setting of possible medical error. When patients die unexpectedly during the course of medical care, such deaths can be classified based on the presence or absence of medical error. When it is unclear if medical error is present or how medical care rendered and unexpected patient death are related, autopsy becomes an important tool for detailing the cause and manner of death. Nurses, as mentioned, are often central to cases of medical error leading to patient death. When a patient dies unexpectedly, they are often the provider who spends the most time talking with the patient's family and potentially play a roll in helping families decide about autopsy. However, autopsy and error reporting to the police are responsibilities charged directly to physicians, not to nurses. This is likely the reason many nursing schools don't cover the topics of autopsy or reporting patient deaths to the police.

Likewise, sharing information regarding adverse events with other institutions for the purpose of learning and error prevention is considered the responsibility of risk managers, not to nurses, and thus many nursing schools do not cover this topic.

Failure mode and effects analysis is an advanced and somewhat in-depth topic that requires expertise and experience to teach effectively. Lack of nursing educators trained in this area may be the reason many nursing schools do not cover this topic.

When compared to public schools, statistically greater private schools covered the topic of root cause analysis while other topics were covered equally. While the reason for this is unclear, perhaps the educational goals of private and public schools differ; we do not know of any differences in incentives (e.g. compensation payout) that differ between private and public schools; exploring these reasons could be a topic of future research.

### For the future

This survey research suggests which topics are covered and which are not in regards to patient safety education. We are providing the results of this study to all Japanese nursing schools, the MEXT, and the MHLW. The topics that are not covered will be of particular relevance in creating curricular guidelines. Once a guideline is created, further research should be conducted to monitor for change in the medical safety curricula.

### Limitations

Our study has several limitations. First, the results are derived from a cross-sectional survey that is subject to bias and the good-subject effect. Second, our participation rates were modest and it is possible that non-responders differ significantly from responders, namely non-responders may be more likely to lack a medical safety program. However, it should be noted that our response rates are not atypical for postal survey research of healthcare professionals and nurses [32-34]. If we assume that all non-responders do not cover patient safety education, we could estimate that only 39% of nursing schools cover the topic. On the other hand, it may be possible that non-responders did not have any one person leading patient safety but do include patient safety education within the curricula. Finally, the validity of our assessment has not been verified. As respondents are simply giving their perceptions of the nursing school's curriculum, this may or may not truly reflect the curricula absolutely.

## Conclusions

This study elucidates the current state of patient safety education in Japanese nursing schools, indicating that most Japanese nursing schools teach patient safety. This demonstrates that national inclusion of patient safety into the nursing curricula is an achievable goal. Nonetheless, we believe that much work still needs to be done to improve the curricula, including the addition of important topics such asthe concepts of apology, management of medical personnel following an adverse event, and autopsy. As errors will happen [4], an important part of medical safety is responding to adverse events [24]. The results of this study are being provided to nursing schools, the MEXT and, the MHLW in hopes that this will aid in the challenging task of creating a safer environment for patients through nursing school education.

## Competing interests

The authors declare that they have no competing interests.

## Authors' contributions

SM conceived of the study and designed the study. EK SM participated in the statistical analysis. SM JS KE also contributed to the manuscript. All authors read and approved the final manuscript.

## Supplementary Material

Additional file 1 **Patient Safety Education Topics by Category and Topic as Reported by Participants in the 2010 National Survey of Safety Education at Japanese Nursing Schools**. For statistical analysis, the chi-square test or Fisher's exact test was used; blank responses were excluded. † P < 0.05 comparing public and private nursing schools. Click here for file
