# Supplementary material for: Patient safety education at Japanese nursing schools: results of a nationwide survey
Source: BMC Res Notes. 2011 Oct 17;4:416. doi: 10.1186/1756-0500-4-416 (PMC3215394; doi:10.1186/1756-0500-4-416)
Supplement: Additional file 1 — Patient Safety Education Topics by Category and Topic as Reported by Participants in the 2010 National Survey of Safety Education at Japanese Nursing Schools. For statistical analysis, the chi-square test or Fisher's exact test was used; blank responses were excluded. † P < 0.05 comparing public and private nursing schools. [file 1756-0500-4-416-S1.DOC]

Additional file 1. Patient Safety Education Topics by Category and Topic as Reported by Participants in the 2010 National Survey of Safety Education at Japanese Nursing Schools

|  |  | Public (N=41) | Private (N=29) | Total (N=70) |
| --- | --- | --- | --- | --- |
|  |  | n (%) | n (%) | n (%) |
| **Hospital safety management** | |  |  |  |
|  | Institutional near-miss/adverse event reporting | 31(75.6) | 19(65.5) | 50(71.4) |
|  | Principles of patient safety | 26 (63.4) | 21 (72.4) | 47(67.1) |
|  | Committee for patient safety | 26 (63.4) | 17 (58.6) | 43(61.4) |
|  | Patient safety officer | 26(63.4) | 16(55.2) | 42(60.0) |
|  | Staff orientation for patient safety | 23(56.1) | 12(41.4) | 35(50.0) |
|  | Department of patient safety | 19(46.3) | 10(34.5) | 29(41.4) |
|  | Patient relations (patient feedback) | 16(39.0) | 11(37.9) | 27(38.6) |
|  | Investigation committee for adverse events | 14(34.1) | 10(34.5) | 24(34.3) |
|  | Reporting to Japanese Council for Quality Health Care [1] | 12(29.3) | 12(41.4) | 24(34.3) |
| **Medical error theory** | |  |  |  |
|  | Human factors | 34(82.9) | 26(89.7) | 60(85.7) |
|  | Theories and models (Swiss Cheese Model, Heinrich’s Law) | 31(75.6) | 24(87.8) | 55(78.6) |
|  | System factors | 28(68.3) | 24(82.8) | 52(74.3) |
|  | Work environment | 27(65.9) | 21(72.4) | 48(68.6) |
| **Practical safety** | |  |  |  |
|  | Verifying patient identity | 34(82.9) | 26(89.7) | 60(85.7) |
|  | Double-checking | 35(85.4) | 25(86.2) | 60(85.7) |
|  | Communication with senior stuffs | 32(78.0) | 24(82.8) | 56(80.0) |
|  | Standardizing procedures | 27(65.9) | 23(79.3) | 50(71.4) |
|  | Identifying risks and developing prevention strategies | 27(65.9) | 22(75.9) | 49(70.0) |
|  | Object pointing with verbal confirmation | 26(63.4) | 21(72.4) | 47(67.1) |
|  | Reading back verbal orders | 23(56.1) | 18(62.1) | 41(58.6) |
|  | Communication of near-miss/adverse events internally | 20(48.8) | 15(51.7) | 35(50.0) |
|  | Modifying drug names | 17(41.5) | 17(58.6) | 34(48.6) |
|  | Patient cooperation | 17(41.5) | 17(58.6) | 34(48.6) |
|  | Fail-safe systems | 13(31.7) | 15(51.7) | 28(40.0) |
|  | Concept of fool-proof | 14(34.1) | 14(48.3) | 28(40.0) |
|  | Coherence of documentation of adverse events | 18(43.9) | 10(34.5) | 28(40.0) |
|  | Appropriate documentation of adverse events | 15(36.6) | 11(37.9) | 26(37.1) |
|  | Confirming orders | 10(24.4) | 8(27.6) | 18(25.7) |
| **Error analysis** | |  |  |  |
|  | Software, Hardware, Environment, and Liveware (SHEL) Model | 18(43.9) | 15(51.7) | 33(47.1) |
|  | Root Cause Analysis　† | 10(24.4) | 14(48.3) | 24(34.3) |
|  | 4M-4E | 12(29.3) | 11(37.9) | 23(32.9) |
|  | Failure Mode and Effect Analysis (FMEA) | 4(9.8) | 6(20.7) | 10(14.3) |
| **Management of adverse events** | |  |  |  |
|  | Emergency protocols | 19(46.3) | 14(48.3) | 33(47.1) |
|  | Documentation | 18(43.9) | 14(48.3) | 32(45.7) |
|  | Formulating prevention strategies | 15(36.6) | 16(55.2) | 31(44.3) |
|  | Definition of terms | 16(39.0) | 14(48.3) | 30(42.9) |
|  | Patient communication | 15(36.6) | 12(41.4) | 27(38.6) |
|  | Hospital investigation | 13(31.7) | 9(31.0) | 22(31.4) |
|  | Transparency/public disclosure | 10(24.4) | 12(41.4) | 22(31.4) |
|  | Preservation of evidence | 11(26.8) | 10(34.5) | 21(30.0) |
|  | Apology | 11(26.8) | 10(34.5) | 21(30.0) |
|  | Management of medical personnel involved in the adverse event | 10(24.4) | 8(27.6) | 18(25.7) |
|  | Analyzing medical errors | 10(24.4) | 8(27.6) | 18(25.7) |
|  | Sharing adverse events with other institutions for learning | 6(14.6) | 8(27.6) | 14(20.0) |
|  | Reporting unnatural deaths to the police | 7(17.1) | 6(20.7) | 13(18.6) |
|  | Recommending autopsy | 3(7.3) | 5(17.2) | 8(11.4) |
| **Autopsy** | |  |  |  |
|  | Clinical autopsy | 8(19.5) | 5(17.2) | 13(18.6) |
|  | Judicial autopsy | 6(14.6) | 2(6.9) | 8(11.4) |
|  | Administrative autopsy | 5(12.2) | 2(6.9) | 7(10.0) |
|  | Model Project for healthcare-associated patient deaths [2] | 3(7.3) | 3(10.3) | 6(8.6) |
| **Legal and societal responsibilities** | |  |  |  |
|  | Criminal prosecution | 32(78.0) | 22(75.9) | 54(77.1) |
|  | Civil liabilities | 32(78.0) | 19(65.5) | 51(72.9) |
|  | Administrative penalties on the individual | 29(70.7) | 20(69.0) | 49(70.0) |
|  | Societal responsibilities | 23(56.1) | 20(69.0) | 43(61.4) |
|  | Administrative penalties on the institution/system | 22(53.7) | 13(44.8) | 35(50.0) |

For statistical analysis, the chi-square test or Fisher’s exact test was used; blank responses were excluded.
† P<0.05 comparing public and private nursing schools.

1. Japan Council for Quality Health Care: **Project to Collect Medical Near-Miss/Adverse Event Information 2009 Annual Report**; 2010.

[http://www.med-safe.jp/pdf/year_report_english_2009.pdf]

1. **Model Project for healthcare-associated patient deaths** (in Japanese)

[http://www.medsafe.jp/index.html]
